# Oxidative/Antioxidative Status in Patients after Myocardial Infarction and in Those without Cardiovascular Event Depending on Anthropometric Factors Defining Body Weight

**DOI:** 10.3390/ijerph16214077

**Published:** 2019-10-23

**Authors:** Grzegorz Józef Nowicki, Barbara Ślusarska, Andrzej Prystupa, Maciej Polak, Maria Czubaj-Kowal, Ewa Rudnicka-Drożak

**Affiliations:** 1 Department of Family Medicine and Community Nursing, Medical University of Lublin, Staszica 6 Str., PL-20-081 Lublin, Poland; basiaslusarska@gmail.com; 2 Department of Internal Medicine, Medical University of Lublin, Staszica 16 Str., PL-20-081 Lublin, Poland; aprystup@wp.pl; 3 Department of Epidemiology and Population Studies, Jagiellonian University Medical College, Grzegórzecka 20 Str., PL-31-531 Cracow, Poland; maciej.1.polak@uj.edu.pl; 4 Department of Pediatrics, St. Żeromski Hospital of Cracow, Na Skarpie 66 Str., 31-913 Cracow, Poland; martacz58@gmail.com; 5 Department of Family Medicine, Medical University of Lublin, Langiewicza 6A Str., PL-20-032 Lublin, Poland; edrozak@poczta.onet.pl

**Keywords:** oxidative status, antioxidant status, oxidative stress, cardiovascular diseases, overweight, obesity

## Abstract

Obesity is one of the factors leading to the development of atherosclerosis. This metabolic disorder is associated with an increased production of reactive oxygen species, which affect the oxidative stress levels. The aim of this study was to evaluate oxidative/antioxidative status and to investigate the correlation between redox markers and anthropometric parameters and body composition in adult patients after myocardial infarction and in individuals without a cardiovascular event in the past. Descriptive data on socio-demographic, clinical, and anthropometric features and blood samples were collected and categorized into two equal groups: after myocardial infarction (study group (SG), *n* = 80) and without a cardiovascular event (control group (CG), *n* = 80). The oxidative/antioxidative status was assessed in plasma on the basis of total oxidative/capacitive status (PerOx), total antioxidative status/capacity (ImAnOx), and oxidized low-density lipoprotein (oxLDL). The oxLDL was significantly higher in the CG group compared to the SG group (*p* = 0.02). No significant differences were found with regard to PerOx and ImAnOx values between the groups studied. A significant positive correlation between PerOx and percentage of adipose tissue (FM%) and body adiposity index (BAI) was found in the two studied groups. ImAnOx significantly positively correlated with visceral adiposity indexes(VAIs) in SG and FM% in CG. OxLDL negatively correlated with body mass index and waist to hip circumference ratio in CG. The total oxidative/antioxidative status is related to the amount of adipose tissue and the BAIs of the subjects. It was observed that it correlates more frequently with the visceral distribution of body fat.

## 1. Introduction

Cardiovascular diseases (CVDs) constitute a major health problem worldwide, and reducing mortality due to these diseases and their consequences is one of the important priorities for healthcare industries in the developed countries [1]. Atherosclerosis underlies most CVDs and is a disease that is characterized by multifactorial etiopathogenesis [2]. The origin and course of atherosclerosis is strongly influenced by oxidative stress in blood vessel walls, which is defined as an imbalance between the production of reactive oxygen species (ROS) in cells and the antioxidant capacity of the organism [3]. Oxidative stress is responsible for causing oxidative damage to lipids, proteins, and nucleic acids, and results in the modification of their structures and functional properties [4,5]. ROS include small and highly reactive compounds—such as free radicals, containing an unpaired electron;e.g., a peroxide anion (O_2_^−^) anda hydroxyl radical (OH·)—and hydrogen peroxide (H_2_O_2_), which is not a free radical. Physiologically, ROS participate in many important cellular processes, such as growth, proliferation, differentiation, apoptosis, gene expression regulation, protein phosphorylation, and immune defense mechanisms. However, in the case of oxidative/antioxidative balance disorders, ROS are produced in excess and contribute to many pathological processes involved in the formation and progression of atherosclerosis. There are three types of ROS activities. Oxidative stress induces strong oxidation and causes damage to proteins, lipids, phospholipids, cell membranes, and DNA, thus contributing to cell dysfunction and destruction. ROS produced in excess react with nitric oxide (NO), which impairs the bioavailability of NO, and thus weakens the vasodilatation function of the endothelium. Moreover, ROS modulate the activity of many cellular proteins and signal pathways (redox signaling), thus inducing specific acute or chronic changes in the phenotype and functioning of the cells [6]. The sources of ROS in the cell include: respiratory chain, xanthine oxidase, uncoupled endothelial NO synthase, cyclooxygenase, myeloperoxidase, andlipoxidase [7]. Among these sources, enzymes belonging to the NADPH oxidase family seem to be the key enzymes responsible for ROS production in the cells of vascular walls [6]. Many studies have demonstrated that oxidative stress is associated with CVDs and that many atherosclerosis risk factors influence the level of oxidative stress [8,9,10].

Obesity, i.e., an excessive accumulation of adipose tissue, is considered to be one of the CVD risk factors, and at the same time, a factor influencing oxidative stress level [11,12]. Adipose tissue, the organ that affects energy homeostasis of the organism, is mainly composed of adipocytes and other cells (e.g., fibroblasts, fibroblastic pre-adipocytes, and endothelial and immune cells) that secrete hormones and cytokines (adipokines or adipocytokines), which subsequently exert endocrine, paracrine, and autocrine effects in the organism. Production of excess energy results in energy accumulation in adipocytes, hypertrophy of adipose tissue, and its hyperplasia. Obesity alters metabolic and endocrine functions of adipose tissue and leads to increased release of hormones, fatty acids, and proinflammatory molecules that contribute to obesity-related complications [13]. In physiological states, and even more so in pathological ones, adipokines induce ROS production, affecting oxidative stress formation. Several mechanisms are involved in the development of oxidative stress in obese individuals, and the mechanisms related to oxidative stress formation are strongly associated with pro-inflammatory processes that cause endothelial damage and excessive formation of free radicals. The presence of excessive adipose tissue was found to be a source of pro-inflammatory cytokines, including tumor necrosis factor alpha, interleukin (IL)-1β and IL-6 [14], C-reactive protein, leptin, and resistin [15].

The formation of ROS and NO is an inseparable phenomenon accompanying biochemical changes occurring in the human body, which under hemostasis conditions have developed mechanisms to protect biomolecules against the harmful effects of free radicals. Protective functions are performed by enzymes like peroxide dismutase, catalase, and glutathione peroxidase; water; fat-soluble antioxidants (e.g., glutathione, ascorbate (vitamin C), α-tocopherol (vitamin E), and β-carotene); and endogenous antioxidants (e.g., albumin, bilirubin, and uric acid) [16,17]. Studies show that mitochondria of white adipose tissues, especially in obese individuals, are the main sites of ROS generation with participation of an increased expression of NDPH and decreased expression of antioxidant enzymes [18]. Oxidative damage to important cellular structures is one of the factors responsible for the development of obesity-related complications such as atherosclerosis, hypertension, insulin resistance, and type 2 diabetes [19,20].

Many markers are currently used to evaluate oxidative and antioxidative status in an individual. These include total oxidant capacity (TOC), total antioxidant capacity (TAC); oxidative stress index, which expresses the TOC/TAC ratio; and oxidized low-density lipoproteins (oxLDLs), which are lipid peroxidation metabolites [21,22,23,24]. Although there are many studies on oxidative stress in obese adults in the literature, to our knowledge there are no reports on this topic in the group of patients who were hospitalized during the beginning phase of cardiovascular rehabilitation program following myocardial infarction and were continuing with motor, diet, and pharmacological therapy. Therefore, the aim of this study was to assess the oxidative/antioxidative status and to investigate the correlation between redox markers and anthropometric parameters and body composition in adult patients after an episode of myocardial infarction and in those who did not suffer from a cardiovascular event previously. The first hypothesis was that people without a cardiovascular event in the past have higher levels of antioxidative markers and lower levels of oxidative ones. The second hypothesis was that the oxidative/antioxidative status is associated with obesity parameters, especially those describing the visceral distribution of adipose tissue.

## 2. Materials and Methods

### 2.1. Study Design and Population

Based on published research [4,10,21] the study was powered to detect the significant correlation equal to 0.33 between oxidative status and anthropometric factors describing the composition of body weight. In order to detect the postulated correlation with 80% power using a two-sided, 5% level test, a minimum of 70 participants in each group were required for the final analysis. A cross-sectional study was conducted during the period from August to December 2017 among 160 adults divided into two equal groups: study group and control group.

Study group (SG) included patients, who after suffering from a myocardial infarction were hospitalized during the early period of cardiac rehabilitation (up to 14 days after discharge from full revascularization) and who continued with physiotherapy, dietary, and pharmacological therapy in the “Uzdrowisko Nałęczów” S.A. Health Resort in Nałęczów and in the Railway Health Resort Hospital in Nałęczów (in eastern Poland). The study included patients from consecutive rehabilitation periods, which were held at 21 or 28 day intervals. All patients in this group were treated with primary percutaneous coronary intervention after the first myocardial infarction. The criteria for inclusion in the SG were as follows: age 40–65 years;being hospitalized after myocardial infarction;in active employment;and provide written consent to participate in the study. Exclusion criteria were as follows: renal failure, cancer, history of pulmonary or rheumatic diseases, and ages under 40 years and over 65 years. Other exclusion criteria included factors that may influence oxidative status, such as infection (e.g., respiratory tract infections) and antioxidant vitamin intake.

The control group (CG) included professionally active adults without a cardiovascular event in the past but reporting for follow-up examinations to the occupational physician as part of periodic examinations. Respondents were recruited from the Provincial Center for Occupational Medicine of the Center for Prophylaxis and Therapy in Lublin (eastern Poland). The criteria for inclusion in the study were as follows: age between 40 and 65 years, no history of a cardiovascular event, low 10-year risk of a cardiovascular event (SCORE index < 5) [25], professional status (working person), no chronic diseases (renal failure, cancer, rheumatism, and lung diseases), no cardiovascular ailments that could suggest the existence of atherosclerotic CVD, no acceptance of preparations modifying the risk of atherosclerosis, and receiving no therapy for hypertension, pre-diabetes mellitus, and hypercholesterolemia. The exclusion criteria were as follows: active infection, intake of drugs or dietary supplements that may affect oxidative status (e.g., vitamins), and long-term use of fruit and vegetable diets only [26].

During the initial visit, all participants were investigated using an extensive questionnaire, interview, physical examination, and additional tests (anthropometric measurements), and the next day after the patient was prepared (12 h of fasting), laboratory tests were carried out.

The research project received a positive opinion from the Bioethics Committee at the Medical University of Lublin (KE-0254/197/2017) and was conducted in accordance with the Helsinki Declaration. All respondents were presented with the purpose of the study and then asked for written consent to participate in the study.

### 2.2. Blood Sample

Blood samples were taken from the elbow vein of fasting subjects in the morning (07:00–09:00) after an overnight rest in two tubes with clotting activator and separating agent (granules) and were delivered to the laboratory within 1 h. Samples were stored at 4 °C until the blood sample was delivered to the laboratory. The plasma was separated by centrifugation at a rate of 3000 rpm for 10 min. The serum from one tube was used for biochemical assays, while the serum from the second sample was transferred to Eppendorf tubes immediately after centrifugation, then frozen at −80 °C, and stored until the markers of oxidative/antioxidative status were determined.

Centrifuged serum from one of the tubes was used to determine the lipid profile (total cholesterol (TC), triglycerides (TG), high-density lipoprotein cholesterol (HDL-C)), serum glucose, and creatinine using standard laboratory methods. Low-density lipoprotein cholesterol was calculated from the Friedewald formula (when TG < 400 mg/dL), estimated glomerular filtration rate from the Cockcroft-Gault formula, and non-HDL from the non-HDL formula = TC [mg/dL] − HDL-C [mg/dL] [27].

### 2.3. Anthropometric Measurements

Anthropometric measurements of body height and weight were performed on all patients. Height was measured with the accuracy of 0.1 cm using an altimeter and body weight was measured without shoes and top-wear using a platform scale with the accuracy of 0.1 kg. Then, the body mass index (BMI) index, defined as body weight in kilograms (kg) divided by the height in square meters (kg/m^2^), was calculated for all subjects [28]. A non-flexible measuring tape was used to measure the waist circumference (WC), between the lower edge of the ribbed arch and the upper comb of the hip bone, and the hip circumference (HC), at the level of the curve of the larger femur. Both measurements were taken in a standing position. Then, the ratios of waist to hip circumference (WHR) and waist to height circumference (WHtR) were calculated [29].

The percentage of adipose tissue content (FM%) was assessed using an electrical bioimpedance method with a body composition analyzer (OMRON Model BF306) according to the manufacturer’s algorithm.

Visceral adiposity indexes (VAIs) and body adiposity index (BAIs) were calculated for all respondents on the basis of anthropometric measurements and biochemical results, but VAIs were calculated separately for women and men. VAI for women = (WC/(36.58 + (1.89 × BMI))) × (TG / 0.81) × (1.52/HDL); VAI for men = (WC/(39.68 + (1.88 × BMI))) × (TG/1.03) × (1.31/HDL) [30], where TG represents triglycerides and HDL represents high-density lipoprotein. BAI = (HC (cm)/height (m) × 1.5) − 18 [31,32].

### 2.4. Determination of Oxidative/Antioxidative Status Markers

Oxidative stress markers such as total oxidative/capacitive status (PerOx (TOS/TOC)) and total antioxidative status/capacity (ImAnOx (TAS/TAC)) were evaluated using the photometric technique. The quantitative immunoenzymatic method was used to measure oxidized LDL(oxLDL). The determinations were performed by ELISA tests using the original reagents (ImmunDiagnostik, Bensheim, Germany).

### 2.5. Statistical Analysis

Continuous variables are summarized by means ± standard deviations or by medians and interquartile ranges (q1–q3). Categorical data are presented as numbers and percentages (%). Comparison of variables of interest between clinical and control study groups was done using *t*-tests or Mann–Whitney tests for continuous variables, and we usedthe chi-squared test for categorical variables. Due to skewness in the antioxidant values of oxLDL, PerOx values (right-skewed) were natural logarithm transformed and the ImAnOx values (left-skewed) were squared. Associations between log oxLDL, log PerOx, and the square of ImAnOx and anthropometric variables were assessed by linear regression. Multiple liner regression models were performed to take into account the influence of gender (model I), age (model II), or smoking status (model III) on tested relationships. All analyses were performed in the whole sample and in study and control groups. Statistical analyses were performed using IBM SPSS Statistical for Windows, version 25.0 (IBM Corporation, Armonk, NY, USA). A *p*-value < 0.05 was considered to be statistically significant.

## 3. Results

### 3.1. Characteristics of Participants

Table 1 presents the characteristics of the studied groups. The study involved 160 patients who were divided into two groups. The mean age in SG was 53.34 ± 4.74 years, and in CG it was 49.25 ± 6.23 years. No significant differences were observed in terms of gender, marital status, and family history of CVD in the groups studied (*p* > 0.05). Taking weight parameters into account, the SG was characterized by significantly higher BMI, WHR, WHtR, and VAI values (*p*< 0.05) compared to those observed in CG.

### 3.2. Oxidative/Antioxidative Status

Table 2 shows the oxidative/antioxidative status in the groupsstudied. There were no significant differences in PerOx levels between SG and CG patients, although the median value of this parameter was higher in CG and amounted to 718.01 µmol/L compared to SG, in which the median value was 699.44 µmol/L. Similarly, no significant differences were observed in the ImAnOx levels between the groups (*p* = 0.35). However, the higher median of this parameter was characteristic for SG (293.8 µmol/L) compared to CG (276.09 µmol/L). On the other hand, oxLDL was significantly higher in the group of patients without a cardiovascular event than in the group of patients after myocardial infarction (*p* = 0.02).

**Figure 1:**
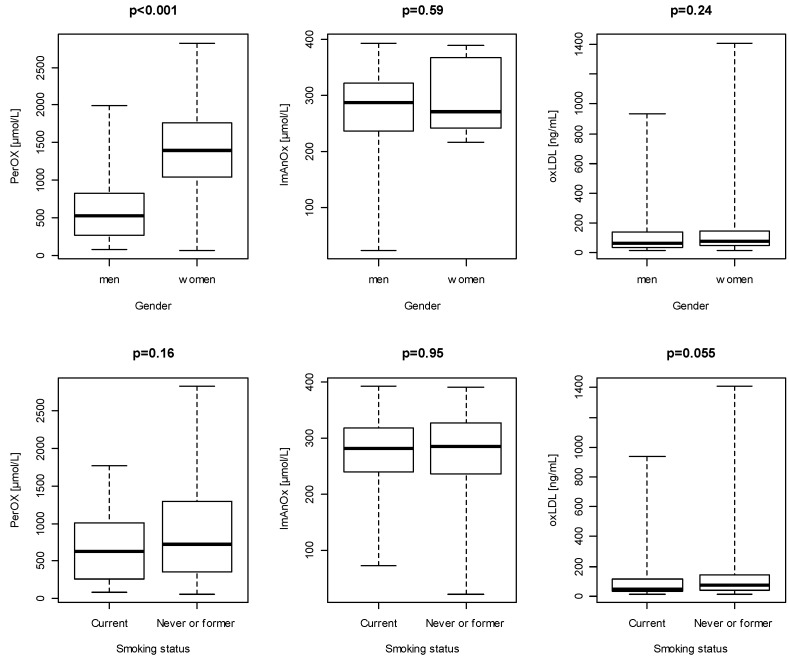
Comparison of the distribution of oxidation/antioxidative status parameters between men and women and smoking status (*n* = 160). PerOx: total oxidative status/capacity; ImAnOx: total antioxidative status/capacity; oxLDL: oxidized low-density lipoprotein.

Figure 1 illustrates a comparison of the oxidative/antioxidative status distribution in gender groups and depending on the smoking status of the entire sample. Gender significantly differentiated the overall oxidation/capacity (PerOx) status; higher values were found in women compared to men (*p* < 0.001). In other cases, no significant differences were found.

### 3.3. Correlation between Oxidative/Antioxidative Status and Anthropometric Measures

Table 3, Table 4 and Table 5 show the relationships between oxidative/antioxidative status and anthropometric factors. In simple linear regression models, significant positive associations between PerOx and FM% (SG: b = 0.043, *p* < 0.001; CG: b = 0.042, *p* < 0.001) and BAI (SG: b = 0.043, *p* < 0.001; CG: b = 0.058, *p* = 0.01) were found in the two studied groups and among all the respondents (FM%: b = 0.042, *p* < 0.001; BAI: b = 0.048, *p* < 0.001). Significant negative associations between PerOx and WHR (b = −2.63, *p* = 0.01) and VAI (b = −0.184, *p*< 0.001) were observed in CG and in the whole examined population (WHR: b = −1.769, *p* = 0.01; VAI: b = −0.103, *p* = 0.01). Moreover, in CG group, PerOx was negatively related with WC (b = −0.02, *p* = 0.02). ImAnOx TAS/TAC were significantly and positively related with VAI (b = 6176.0, *p*< 0.001) in SG, with FM% (b = 1431.0, *p* = 0.04) in CG, and with these two parameters in the whole group (VAI: b = 3754.0, *p* = 0.02; FM%: b =762.0, *p* = 0.049). OxLDL were negatively related with WHR (b = −2.37, *p* = 0.04) in CG and with WHR (b = −1.969, *p* = 0.01) in the whole examined group. After adjustment for age or smoking status, the results were similar to those obtained in the simple linear regression models (Table 1, Table 2 and Table 3). However, after adjustment for gender, the relationships between PerOx and WHR, FM%, WC, and BAI lost statistical significance.

## 4. Discussion

The relationship between oxidative stress and CVD is of immense interest to many researchers. It was demonstrated that both excessive oxidative stress and inadequate antioxidative defense mechanisms may cause an early onset of CVD [33]. Increased oxidative stress markers act in synergy with standard risk factors for CVDs [34,35]. In this cross-sectional study, we evaluated the oxidative/antioxidative status in parallel groups and examined the correlation between redox markers and anthropometric parameters and body composition in the group of adult patients undergoing cardiac rehabilitation after suffering from myocardial infarction and in individuals who had not suffered from a cardiovascular event in the past. This is probably the first study to evaluate the oxidative/antioxidative status of a group of people who enrolled in a cardiovascular rehabilitation program following myocardial infarction and in those without a cardiovascular event, during the subclinical development of atherosclerosis, and to identify which patients required primary prevention (CG) or secondary prevention (SG) based on the risks of cardiovascular complications. The results of our study did not confirm the first hypothesis. The markers of oxidative/antioxidative status were better indicated in SG than in CG. The second hypothesis was confirmed because our study proved that visceral fat distribution is correlated with oxidative/antioxidative status, so management should place particular emphasis on weight reduction.

Our study showed that lipid peroxidation (oxLDL) was much stronger in CG than in SG patients. The result we obtained were completely different from the results obtained by Dominguez-Rodriguez et al. [36], who evaluated nighttime oxLDL and melatonin levels in patients with acute coronary syndrome (ACS) and healthy subjects without symptomatic atherosclerosis. In this study, the group of patients with ACS had higher levels of oxLDL and lower levels of melatonin than CG patients. However, in Renko et al.’s [37] study conducted on a group of 120 men after myocardial infarction, whichhad 250 men in the CG, no significant differences between groups were found in the oxLDL level. The results of our study wereobtained in a group of patients who, after suffering from myocardial infarction were receiving early cardiological rehabilitation, including physiotherapy, pharmacotherapy, and dietary treatment. As the literature indicates, the occurrence of myocardial infarction leads to the modification of lifestyle-related behaviors to more pro-healthy ones, especially in the context of dietary changes [38]. The change of the diet tothe one containing less fat resulted in a decrease in oxLDL levels in the group consisting of obese women, which was demonstrated by Elizabeth et al. [39] in their study, as well as in the patients who participate regularly in Nordic walking, in the study by Cebula et al. [40]. Moreover, higher oxLDL levels in CG in our study may be associated with higher levels of LDL-C in this group, because the processes involved in the modification of this cholesterol fraction play a key role in the formation of atherosclerotic plaque [41]. The above arguments may also influence the results obtained with respect to other markers of oxidative status, such as PerOx, which was lower in SG, and antioxidative status, such as ImAnOx, which was higher in CG, although these differences were not statistically significant (*p* > 0.05). In our study, CG was characterized by higher blood lipid indices compared to SG (TC, *p* < 0.001; LDL-C, *p* < 0.001; and non-HDL, *p* < 0.001). The available literature data indicate a close correlation between the development of atherosclerotic plaque, determined by the thickness of the intima-media complex, and the concentration of not only TC, but also its individual lipid fractions in blood [42,43,44,45]. A selection of respondents for CG was conditioned by the non-administration of preparations modifying the risk of atherosclerosis; therefore, it can be assumed that high lipid levels correlate with the development of atherosclerotic plaque, and the changes in vascular endothelium are reflected in oxidative/antioxidative disorders.

Drugs taken by the patient are very important for determining the level of oxidative stress. The respondents after myocardial infarction were staying in the Health Resort Hospital and each of the respondents took their medicines systematically. Some of the drugs are known to influence the level of oxidative stress. Aspirin, statins, angiotensin converting enzyme II (Ang II) inhibitor, and metformin are commonly used in the secondary prevention of cardiovascular events and treatment of concomitant diseases; they also exhibit many pleiotropic effects [46]. The Paseban et al.’s [47] study showed that the combined use of the above-mentioned drugs enhances their antioxidant effect. Aspirin has an antioxidant effect by reducing the production of free radicals, such as peroxide, and prevents a decrease in the activity of antioxidant enzymes (catalase and peroxide dismutase) [48]. Statins, due to their antioxidant activity by inhibition of NAD(P)H oxidase and active exchange of free radicals [49,50,51], reduce chronic inflammation [52], and thus reduce oxidative stress [53]. Ang II inhibitor may selectively reduce Ang II, endothelin, and oxidative stress levels, which may potentially play a role in the lowering of blood pressure [54]. Moreover, in our sample, 24% of SG patients are diabetics. Metformin is the first hypoglycemic drug used in the treatment of patients with type 2 diabetes, and it has been shown to reduce ROS by enhancing the activity of antioxidant enzymes [55].

Studying the relationship between gender and oxidative/antioxidant status is vital, as oxidative stress is a factor in the development of numerous diseases, including cardiovascular diseases, and at the same time, these diseases occur differently in men and women.It was proven that oxidative stress was lower in male rats than in females [56].There was observed as a significant difference in the PerOx (TOS/TAC) distribution between males and females in the presented research (Figure 1). Higher values were determined in women. Furthermore, significantly higher values of WC, WHR, FM%, and BAI characterized female participants (data non shown). The existence of the above significant relationships may explain the loss of relationships between PerOx (TOS/TAC), WHR, FM%, WC, and BAI after adjusting for gender. Ide et al. [57] showed that biomarkers of oxidative stress in vivo were higher in young men than in women of the same age. In other studies, it was observed that ROS production was higher in blood vessel cells in men than in women [58], and women were found to have the greater antioxidant potential [59]. Other studies indicate that there is a difference in the expression and/or activity of antioxidant enzymes between men and women. These enzymes are present in various body tissues. In regard to SOD, there is no uniform consensus on gender differences, although it is suggested that there may be variances in different tissues. Chen et al. [60] showed that the level of SOD activity in brain tissue and lungs was higher in female mice, but there were no significant differences in the level of SOD activity between females and males in the kidney and heart. However, Barp et al. [56] established that female rats had a higher level of SOD activity in the heart than males. Interestingly, they also found that after castration, the level of SOD activity in both male and female rats was significantly reduced compared to the control group. The results of the cited researchers indicate that there may be a relationship between sex hormones and the level of SOD activity. However, some studies have not shown differences in the level of SOD activity between men and women; therefore, there are some differences regarding the association of SOD activity and gender [57,61]. Chan et al. [60] revealed that catalase activity in both female and male mice was the same in the brain, lungs, and heart, but higher in females in a kidney. However, some studies did not demonstrate differences in the level of catalase activity between men and women [56,57,62]. Thus, the studies cited above indicate that gender and sex hormones do not affect catalase activity, and thus, the degradation of hydrogen peroxide [63]. Several studies have shown that GPx activity was lower in women than in men [56,57,60], although there was no significant change in GPx levels after castration, suggesting that sex hormones may not affect GPx [56]. The fact that GPx levels were lower in women seems counterintuitive because women are thought to be less prone to oxidative stress than men. This observation suggests that women possess other mechanisms to protect themselves against oxidative stress. Although there may be some differences in the level of antioxidant enzyme activity between men and women, as discussed earlier, the difference in antioxidant properties is probably due to estrogen [63]. This sex hormone acts as an antioxidant, scavenging free radicals due to the presence of a phenolic hydroxyl group [56]. In the presented research, the overall oxidation/capacitance status (PerOx) was higher in women than in men. The explanations for this result can be seen in the menopausal or postmenopausal period in which the women were examined, which reduced the concentration of estrogens in the studied group.

Obesity is considered to be an important risk factor leading to the development of many diseases, such as ischemic heart disease, diabetes, hypertension, dyslipidemia, stroke, and some types of cancer [64,65]. This metabolic disorder is associated with an increased ROS production and oxidative stress formation. Increased ROS production in obese individuals is associated with an excessive supply of macronutrients in the diet, mitochondrial dysfunction, excessive ROS production at the endoplasmic reticulum level, and inflammatory response [66,67]. Obesity is one of the factors leading to oxidative stress, which in turn leads to atherosclerosis of vessels, and consequently, may lead to myocardial infarction. Amirkhizi et al. [68] conducted a study on obese women and found that obesity, even in the absence of smoking, diabetes, kidney, and liver diseases, it may reduce protective antioxidant mechanisms by increasing systemic oxidative stress.

In our study, anthropometric and physiological measurements (WHR, FM%, VAI, and BAI) significantly correlated with the oxidative/antioxidative status in SG and CG patients, as well as in the entire sample. Moreover, we discovered several significant correlations between VAI and BAI and oxidative/antioxidative status in both groups. In our study, the oxidative/antioxidative status was more often correlated with FM%, VAI, and BAI than with BMI. Although BMI is used to measure overweight and obesity, it does not take into account factors such as body size and adipose tissue distribution. Studies suggest that abdominal obesity is more strongly associated with chronic diseases [69,70], because visceral fat secretes several metabolites that cause chronic diseases [71,72]. Chrysohoou et al. [73] concluded in their study that the total oxidative capacity (TAC) was significantly correlated with the central obesity index rather than with obesity. Visceral fat accumulation (measured by computed tomography), as a factor associated with enhanced oxidative state, was also demonstrated by Araki et al. [74]. The importance of adipose tissue distribution in the context of oxidative stress in obesity was demonstrated by other authors who found a correlation between anthropometric parameters (WC and BMI) and the level of oxidative stress activation [75,76].Similarly, in the presented, original research, a significant relationship was observed between WC and PerOx (TOS/TAC) in CG. Pihl et al. [77] conducted research among former athletes who had ceased their professional activity at least 15 years before joining the study. A control group of 54 males and the investigation group constituted a study population of 114 men. The study population was divided into a clinical group (*n* = 60), former competitive athletes who still lead an active lifestyle, and a control group (*n* = 54) including people who did not practice competitive sports in the past and were not physically active at the time of the study. The cited study indicated that oxidized LDL-C had a significant association with WC but also with BMI and WHR. Interestingly, this relationship was demonstrated in the control group, not in the clinical group, and the correlation analysis among the entire study group showed that those relationships were affected by the level of physical activity. Amirkhizi et al. [78] studied the relationship between oxidative stress and total antioxidant capacity in a group of women with general and abdominal adiposity. Authors established that overweight and obese women were characterized by higher plasma malondialdehyde (MDA) levels compared to normal weight women. In addition, abdominal obesity was found to be significantly associated with increased plasma MDA levels. The authors also established the potential relationship between oxidative stress levels and visceral fat distribution. Total plasma antioxidant capacity (TAC) was impaired in participants with central and visceral obesity, which confirmed increased MDA levels and reduced TAC plasma levels.

### Limitations

Our study has a few limitations. First, it is a cross-sectional study, so neither temporality nor causality can be established. Second is the inclusion of a relatively small number of patients fromasingle center, especially inregard to patients qualified to be included in SG. The third limitation of our study is the use of bioelectrical impedance analysis (BIA), which is an indirect method for the evaluation of body composition. However, comparative studies have shown significant correlations between BIA data and body composition measured by densitometry (dual-energy X-ray absorptiometry (DXA)), which is the golden standard for this type of analysis [79]. Unfortunately, DXA is associated with exposure to X-rays, which limitsthe regular use of this method. The fourth limitation of our study was the non-inclusion of drugs taken by patients in the analysis of oxidative stress levels in particular groups, although the main aim of the study was to assess the parameters related to obesity and oxidative/antioxidative status, but these drugs may have influenced the results of the study.

## 5. Conclusions

To sum up, our study shows that total oxidative/antioxidative status is related to the adipose tissue contents and skewness BAIs of the subjects, but it was observed that it correlates more frequently with the visceral distribution of adipose tissue. Given the significant association of visceral fat distribution with oxidative/antioxidative status, further studies are needed to investigate the impact of fat distribution on the risk of cardiometabolic diseases, especially in conjunction with other common cardiovascular risk factors.

## Figures and Tables

**Table 1:** Baseline characteristics of the study population.

Variables	Study Group (*n* = 80)	Control Group (*n* = 80)	*p*
Demgraphic data:			
Age [years] ^b^	53.34 ± 4.74	49.25 ± 6.23	<0.001
Sex (Female vs. Male) ^a^	22 (27.5) vs. 58 (72.5)	28 (35.0) vs. 52 (65.0)	0.32
Marital status (free vs. in relationship) ^a^	16 (20.0) vs. 64 (80.0)	11 (13.7) vs. 69 (86.3)	0.398
Clinical variables:			
Family history of CVD—on the mother’s side ^a^	46 (57.5)	42 (52.5)	0.634
Family history of CVD—on the father’s side ^a^	49 (61.3)	39 (48.8)	0.153
Diabetes ^a^	19 (24.0)	0 (0.0)	<0.001
Arterial hypertensions ^a^	54 (68.0)	0 (0.0)	<0.001
Smoking ^a^	25 (31.2)	9 (11.2)	<0.001
Anthropometric variables:			
BMI [kg/m^2^] ^b^	28.89 ± 4.91	26.64 ± 4.04	0.002
WC [cm] ^b^	103.9 ± 12.48	93.36 ± 12.50	<0.001
HC [cm] ^b^	105.0 ± 10.83	102.9 ± 7.72	0.15
WHR ^b^	0.99 ± 0.08	0.91 ± 0.09	<0.001
WHtR^b^	0.60 ± 0.07	0.54 ± 0.07	<0.001
FM% ^b^	31.08 ± 7.71	29.65 ± 7.05	0.22
VAI ^b^	2.12 ± 1.56	1.06 ± 0.7	<0.001
BAI ^b^	28.63 ± 6.44	27.79 ± 4.73	0.35
Biochemical parameters:			
Total cholesterol [mg/dL] ^b^	147.81 ± 37.00	221.98 ± 44.22	<0.001
Triglyceride [mg/dL] ^c^	134.52 (103.25–174.35)	102.91 (73.41–141.15)	<0.001
HDL-C [mg/dL] ^b^	46.16 ± 11.50	64.05 ± 19.14	<0.001
non-HDL [mg/dL] ^b^	101.50 ± 34.31	157.90 ± 47.22	<0.001
LDL-C [mg/dL] ^b^	70.24 ± 25.86	134.81 ± 42.37	<0.001
Glucose [mg/dL] ^c^	102.51 (97.01–112.01)	102 (97.51–109.51)	0.78
Creatinine [mg/dL] ^b^	0.85 ± 0.15	0.86 ± 0.18	0.97
eGFR [mL/min/1.73 m^2^] ^b^	93.52 ± 12.56	95.40 ± 13.33	0.35

Date are presented as: ^a^ *n*(%); ^b^ mean ± SD; ^c^ median (Q1–Q3). BMI: body mass index; WC: waist circumference; HC: hip circumference; WHR: waist to hip ratio; WHtR: waist-to-height ratio; FM%: body fat percentage; VAI: visceral adiposity index; BAI: body adiposity index; HDL-C: high-density lipoprotein; LDL-C: low-density lipoprotein.

**Table 2:** Oxidative/antioxidative status markers comparison of studied groups.

Variables	Study Group (*n* = 80)	Control Group (*n* = 80)	*p*
PerOx (TOS/TOC) [µmol/L]	699.44 (379.71–1152.36)	718.01 (298.35–1274.61)	0.77
ImAnOx (TAS/TAC) [µmol/L]	293.8 (246.52–317.92)	276.09 (233.76–333.33)	0.35
oxLDL [ng/mL]	54.25 (36.09–119.34)	75.91 (49.38–143.32)	0.02

PerOx (TOS/TOC): total oxidative status/capacity; ImAnOx (TAS/TAC): total antioxidative status/capacity; oxLDL: oxidized low-density lipoprotein.

**Table 3:** Correlation between oxidative/antioxidative status and anthropometric measures in study group.

Variables	Model	Log PerOx (TOS/TAC) [µmol/L]		Square ImAnOx (TAS/TAC) [µmol/L]		Log oxLDL [ng/mL]	
		b (SE)	*p*	b (SE)	*p*	b (SE)	*p*
BMI	0	0.02 (0.019)	0.300	64 (666.0)	0.310	0.009 (0.021)	0.680
	I	0.014 (0.017)	0.407	714.9 (669.72)	0.289	0.009 (0.021)	0.661
	II	0.015 (0.019)	0.415	735.11 (675.86)	0.280	0.003 (0.021)	0.892
	III	0.018 (0.019)	0.335	711.0 (688.23)	0.291	0.008 (0.021)	0.719
WC	0	0.001 (0.007)	0.86	85.9 (392.9)	0.83	0.002 (0.008)	0.78
	I	0.007 (0.007)	0.33	210.9 (268.3)	0.43	0.002 (0.008)	0.83
	II	0.0003 (0.007)	0.97	259.3 (265.5)	0.33	0.0003 (0.008)	0.97
	III	0.0008 (0.007)	0.91	250.4 (262.9)	0.34	0.002 (0.008)	0.81
WHR	0	−1.43 (1.081)	0.190	60,029.0 (37,916.0)	0.120	−0.999 (1.218)	0.410
	I	1.009 (1.174)	0.393	63,250.68 (45,055.06)	0.164	−1.736 (1.439)	0.234
	II	−1.207 (1.081)	0.268	58,633.78 (38,483.66)	0.132	−0.696 (1.209)	0.567
	III	−1.358 (1.078)	0.211	58,559.73 (38,063.40)	0.128	−0.950 (1.222)	0.439
WHtR	0	1.989 (1.23)	0.110	30,831.0 (43,917.0)	0.480	0.797 (1.396)	0.570
	I	1.523 (1.136)	0.184	34,488.18 (44,262.36)	0.438	0.862 (1.412)	0.543
	II	1.623 (1.250)	0.198	36,756.69 (45,068.24)	0.417	0.234 (1.404)	0.863
	III	1.879 (1.228)	0.130	33,562.45 (44,083.30)	0.449	0.719 (1.403)	0.610
FM%	0	0.043 (0.011)	<0.001	148.0 (426.0)	0.730	0.013 (0.013)	0.350
	I	0.022 (0.015)	0.153	720.46 (597.12)	0.231	0.032 (0.019)	0.092
	II	0.040 (0.011)	<0.001	195.5 (436.75)	0.656	0.0008 (0.013)	0.570
	III	0.041 (0.011)	<0.001	195.97 (430.33)	0.650	0.011 (0.014)	0.407
VAI	0	−0.055 (0.05)	0.280	6176.0 (1647.0)	<0.001	−0.019 (0.057)	0.740
	I	−0.066 (0.046)	0.153	6277.41 (1650.05)	<0.001	−0.018 (0.057)	0.745
	II	−0.056 (0.050)	0.264	6192.41 (1654.50)	<0.001	−0.021 (0.056)	0.706
	III	−0.034 (0.054)	0.537	6506.22 (1779.96)	<0.001	−0.002 (0.061)	0.974
BAI	0	0.043 (0.014)	<0.001	−255.0 (510.0)	0.620	0.018 (0.016)	0.260
	I	0.018 (0.016)	0.260	−67.94 (624.32)	0.914	0.032 (0.019)	0.102
	II	0.039 (0.014)	0.008	−199.5 (538.29)	0.712	0.009 (0.017)	0.567
	III	0.041 (0.014)	0.004	−209.32 (514.55)	0.685	0.017 (0.016)	0.210

Model 0: simple linear regression; Model I: after adjustment for gender; Model II: after adjustment age; Model III: after adjustment for smoking status; b: coefficient form liner regression; SE: standard error;BMI: body mass index; WC: waist circumference; WHR: waist to hip ratio; WHtR: waist-to-height ratio; FM%: body fat percentage; VAI: visceral adiposity index; BAI: body adiposity index; PerOx (TOS/TOC): total oxidative status/capacity; ImAnOx (TAS/TAC): total antioxidative status/capacity; oxLDL: oxidized low-density lipoprotein.

**Table 4:** Correlation between oxidative/antioxidative status and anthropometric measures in control group.

Variables	Model	Log PerOx (TOS/TAC) [µmol/L]		Square ImAnOx (TAS/TAC) [µmol/L]		Log oxLDL [ng/mL]	
		b (SE)	*p*	b (SE)	*p*	b (SE)	*p*
BMI	0	−0.02 (0.025)	0.410	40.0 (1212.0)	0.970	−0.047 (0.025)	0.060
	I	0.010 (0.022)	0.649	735.4 (1226.18)	0.550	−0.043 (0.026)	0.097
	II	−0.023 (0.025)	0.364	281.0 (1207.22)	0.816	−0.047 (0.025)	0.066
	III	−0.022 (0.025)	0.382	−60.9 (1210.39)	0.960	−0.047 (0.025)	0.063
WC	0	−0.02 (0.008)	0.02	239.2 (262.2)	0.36	−0.01 (0.008)	0.08
	I	0.0004 (0.008)	0.96	624.3 (434.5)	0.15	−0.01 (0.009)	0.16
	II	0.02 (0.008)	0.01	235.5 (397.3)	0.56	−0.01 (0.01)	0.09
	III	−0.02 (0.008)	0.02	82.7 (391.5)	0.83	−0.01 (0.008)	0.09
WHR	0	−2.627 (1.015)	0.010	25,426.0 (51,798.0)	0.620	−2.373 (1.109)	0.040
	I	0.682 (1.153)	0.556	149,410.3 (62,602.48)	0.019	−2.548 (1.388)	0.071
	II	−3.203 (1.060)	0.003	58,629.4 (53,765.78)	0.279	−2.606 (1.189)	0.032
	III	−2.619 (1.017)	0.012	26,115.1 (51,603.93)	0.614	−2.337 (1.227)	0.041
WHtR	0	−1.069 (1.463)	0.470	75,144.0 (71,489.0)	0.300	−2.436 (1.508)	0.110
	I	0.340 (1.283)	0.792	109,285.4 (70,845.72)	0.127	−2.219 (1.534)	0.152
	II	−1.327 (1.494)	0.383	−1510.8 (789.92)	0.060	−2.474 (1.552)	0.115
	III	−1.188 (1.470)	0.422	67,786.8 (71,593.44)	0.347	−2.454 (1.515)	0.110
FM%	0	0.042 (0.013)	<0.001	1431.0 (679.0)	0.04	0.004 (0.014)	0.760
	I	0.0005 (0.016)	0.972	881.1 (881.35)	0.321	−0.004 (0.019)	0.618
	II	0.045 (0.013)	0.001	1295.21 (681.61)	0.061	0.004 (0.015)	0.783
	III	0.0418 (0.0138)	0.003	1292.9 (704.02)	0.070	0.003 (0.015)	0.862
VAI	0	−0.184 (0.06)	<0.001	−189.0 (3144.0)	0.950	−0.04 (0.065)	0.540
	I	−0.126 (0.54)	0.022	1361.2 (3158.71)	0.668	−0.028 (0.066)	0.680
	II	−0.184 (0.060)	0.003	−158.7 (3109.71)	0.959	−0.040 (0.066)	0.548
	III	−0.183 (0.060)	0.003	−154.2 (3132.82)	0.961	−0.038 (0.066)	0.560
BAI	0	0.058 (0.02)	0.01	137.0 (1024.0)	0.180	0.007 (0.022)	0.750
	I	0.0001 (0.022)	0.996	192.0 (1257.17)	0.879	−0.010 (0.027)	0.713
	II	0.060 (0.020)	0.004	1203.0 (1021.22)	0.242	0.006 (0.022)	0.772
	III	0.056 (0.020)	0.007	1181.7 (1039.78)	0.259	0.005 (0.022)	0.831

Model 0: simple linear regression; Model I: after adjustment for gender; Model II: after adjustment age; Model III: after adjustment for smoking status; b: coefficient form liner regression; SE: standard error;BMI: body mass index; WC: waist circumference; WHR: waist to hip ratio; WHtR: waist-to-height ratio; FM%: body fat percentage; VAI: visceral adiposity index; BAI: body adiposity index; PerOx (TOS/TOC): total oxidative status/capacity; ImAnOx (TAS/TAC): total antioxidative status/capacity; oxLDL: oxidized low-density lipoprotein.

**Table 5:** Correlation between oxidative/antioxidative status and anthropometric measures in all participants.

Variables	Model	Log PerOx (TOS/TAC) [µmol/L]		Square ImAnOx (TAS/TAC) [µmol/L]		Log oxLDL [ng/mL]	
		b (SE)	*p*	b (SE)	*p*	b (SE)	*p*
BMI	0	0.003 (0.015)	0.850	561.0 (632.0)	0.380	−0.019 (0.016)	0.230
	I	0.011 (0.013)	0.386	646.75 (633.42)	0.309	−0.018 (0.016)	0.252
	II	−0.001 (0.015)	0.920	752.2 (642.87)	0.244	−0.021 (0.016)	0.199
	III	0.003 (0.014)	0.852	561.20 (634.03)	0.377	−0.019 (0.016)	0.241
WC	0	−0.007 (0.005)	0.13	230.7 (215.63)	0.29	−0.09 (0.005)	0.12
	I	0.003 (0.005)	0.47	371.1 (228.6)	0.11	−0.008 (0.06)	0.15
	II	−0.01 (0.005)	0.047	337.7 (224.0)	0.13	−0.01 (0.006)	0.08
	III	−0.007 (0.005)	0.17	231 (2216.8)	0.29	−0.008 (0.005)	0.15
WHR	0	−1.769 (0.669)	0.01	47,355.0 (29,309.0)	0.110	−1.969 (0.742)	0.01
	I	0.740 (0.724)	0.308	99,864.4 (34,768.77)	0.005	−2.426 (0.882)	0.007
	II	−2.174 (0.685)	0.002	61,337.32 (30,174.96)	0.044	−2.203 (0.770)	0.005
	III	−1.656 (0.673)	0.015	48,195.28 (296,499.25)	0.106	−1.841 (0.753)	0.016
WHtR	0	0.443 (0.874)	0.610	59,313.0 (37,497.0)	0.120	−1.167 (0.954)	0.220
	I	0.845 (0.771)	0.275	63,389.59 (37,496.78)	0.093	−0.138 (0.957)	0.236
	II	0.069 (0.916)	0.940	81,717.4 (39,115.65)	0.038	−1.405 (1.005)	0.164
	III	0.481 (0.870)	0.581	59,362.16 (37,630.96)	0.117	−1.103 (0.952)	0.248
FM%	0	0.042 (0.009)	<0.001	762.0 (392.0)	0.05	0.007 (0.01)	0.460
	I	0.011 (0.011)	0.320	764.13 (520.57)	0.144	0.006 (0.013)	0.664
	II	0.041 (0.008)	<0.001	785.61 (391.16)	0.046	0.007 (0.010)	0.470
	III	0.004 (0.009)	<0.001	778.39 (397.22)	0.052	0.005 (0.010)	0.595
VAI	0	−0.103 (0.038)	0.01	3754.0 (1639.0)	0.02	−0.045 (0.041)	0.280
	I	−0.084 (0.033)	0.013	3993.86 (1639.74)	0.016	−0.043 (0.041)	0.039
	II	−0.110 (0.037)	0.003	3999.9 (1640.06)	0.016	−0.047 (0.042)	0.262
	III	−0.094 (0.039)	0.018	4081.83 (1710.957)	0.018	−0.030 (0.043)	0.489
BAI	0	0.048 (0.011)	<0.001	356.0 (517.0)	0.490	0.013 (0.013)	0.320
	I	0.011 (0.013)	0.389	−12.94 (624.18)	0.983	0.012 (0.016)	0.433
	II	0.046 (0.011)	<0.001	437.4 (519.48)	0.401	0.013 (0.013)	0.337
	III	0.046 (0.011)	<0.001	359.78 (522.24)	0.492	0.011 (0.013)	0.403

Model 0: simple linear regression; Model I: after adjustment for gender; Model II: after adjustment age; Model III: after adjustment for smoking status; b: coefficient form liner regression; SE: standard error;BMI: body mass index; WC: waist circumference; WHR: waist to hip ratio; WHtR: waist-to-height ratio; FM%: body fat percentage; VAI: visceral adiposity index; BAI: body adiposity index; PerOx (TOS/TOC): total oxidative status/capacity; ImAnOx (TAS/TAC): total antioxidative status/capacity; oxLDL: oxidized low-density lipoprotein.
